# Strain-dependent and distinctive T-cell responses to HIV antigens following immunisation of mice with differing chimpanzee adenovirus vaccine vectors

**DOI:** 10.1016/j.vaccine.2016.07.028

**Published:** 2016-08-17

**Authors:** S. Herath, A. Le Heron, S. Colloca, S. Patterson, R. Tatoud, J. Weber, G. Dickson

**Affiliations:** a School of Biological Sciences, Royal Holloway, University of London, Egham, Surrey TW20 0EX, UK; b ReiThera Srl, Viale Citta d’Europa 679, 00144 Rome, Italy; c Department of Immunology, Imperial College London, London, UK

**Keywords:** Immunology, HIV, Mouse, Vaccine, IFNγ

## Abstract

*In vivo* vaccination studies are conventionally conducted in a single mouse strain with results, only reflecting responses to a single immunogenetic background. We decided to examine the immune response to an HIV transgene (*gag*, *pol* and *nef* fusion protein) in 3 strains of mice (CBA, C57BL/6 and BALB/c) to determine the spectrum of responses and in addition to determine whether the serotype of the adenoviral vector used (ChAd3 and ChAd63) impacted the outcome of response. Our results demonstrated that all three strains of mice responded to the transgene and that the magnitude of responses were different between the strains. The C57BL/6 strain showed the lowest range of responses compared to the other strains and, very few responses were seen to the same peptide pool in all three strains of mice. In CBA and BALB/c mice there were significant differences in IFNγ production dependent on the adenoviral vector used. Our results suggest that employing a single strain of mouse may underestimate the efficacy and efficiency of vaccine products.

## 1 Introduction

The development of a prophylactic HIV viral vector vaccine has remained challenging due to the absence of immunogenic responses to some viral vectors and the presence of pre-existing neutralising antibody immunity to human adenovirus vectors in a large proportion of the human population. The isolation of new vectors that are able to induce immune responses to the same level as those seen with human adenoviruses and with low seroprevalance of neutralising antibodies in humans, have contributed to the progress of vector vaccine choice [1], [2], [3] though some of these vectors do not mount efficient immune response in animal models [4].

It is known that immune responses to the Gag, Pol and/or Nef proteins of HIV do offer some protection against the virus in several animal models [5], [6], [7], [8]. Most *in vivo* vaccination studies employ the use of a single mouse strain, with the C57BL/6 strain being predominantly used for HIV vaccine studies. We have previously shown that two viral vectors, chimpanzee adenoviruses (ChAd) 3 and 63 bearing a mosaic transgene formed from parts of HIV *gag, pol* and *nef genes*, were able to induce immune responses in C57BL/6 mice [9]. As with most mouse vaccination studies, it is difficult to use these results to predict the efficacy of the vaccine in humans due to the limited immunogenetic variation seen in inbred murine lab strains [10]. In our hands, we found that C57BL/6 responses to our HIV transgene were narrow for both vectors [9]. To compensate for the lack of genetic variability in our animal model, we decided to compare the immune response to our HIV transgene in 3 strains of mice, each representing a different haplotype. We chose the CBA strain, which is *k* restricted, the C57BL/6 strain which is *b* restricted and the BALB/c strain which is *d* restricted. In addition to comparing the immune responses to our HIV transgene of interest, this study also provided the opportunity to determine if there were any differences in responses to the vector employed in the different strains of mice.

## 2 Materials and methods

### 2.1 Mice

CBA, BALB/c and C57BL/6 mice were purchased from Charles River (UK); 6–8 week old females were used. Mice were injected subcutaneously with either 1 × 10^8^ IU of ChAd3 or 1 × 10^9^ IU of ChAd63. Mice were sacrificed 7 days post vaccination. All *in vivo* procedures were performed in accordance with Royal Holloway and Home Office regulations for animal experimentation.

### 2.2 Spleen cell isolation

Splenocyte suspensions were obtained using a glass homogeniser (Fisher) with RPMI containing 10% FBS, 100 IU/ml penicillin and 0.1 mg/ml streptomycin (Gibco). Cells were treated with red cell lysis buffer as directed (Sigma-Aldrich). Live cells were counted using Trypan blue exclusion.

### 2.3 Peptides

Individual HIV-ZM96 Gag overlapping peptides (15-mers overlapping by 10 amino acids) were provided by NIBSC, UK and dissolved in water. Reconstituted individual overlapping peptides for HIV-ZM96 Pol 5′, Nef and Pol 3′ were provided by the International Aids Vaccine Initiative (IAVI). The peptides were pooled into peptide pool matrices and used at a concentration of 1 μg/ml as previously described [9].

### 2.4 IFNγ ELISpot

HIV specific IFNγ T cell responses were determined by restimulation of splenocytes with peptides and quantified by anti-mouse IFNγ ELISpot (Mabtech AB, Sweden) as previously described [9]. Plates were read using an AID ELISpot reader (AutoImmun Diagnostika, Germany).

### 2.5 Statistical analysis

Data were analysed with GraphPad Prism® V6.04 using Multiple parametric independent-measures *T* tests. Significance was reached at *p* < 0.05.

## 3 Results

In our previous work we showed responses in the C57BL/6 mice to an HIV transgene containing elements of *gag*, *pol* and *nef* genes (GPN) delivered by two vectors; ChAd3 and ChAd63 [9]. Our results showed ChAd3-GPN was more immunogenic than ChAd63-GPN and that to produce equivalent IFNγ ELISPOT responses a higher dose of ChAd63-GPN was required. Therefore, in this study different mouse strains were immunised with either 1 × 10^8^ IU of ChAd3-GPN or 1 × 10^9^ IU ChAd63-GPN to determine whether there were differences in responses to the transgenes and vectors.

### 3.1 Strain dependent Gag responses

**Fig. 1:**
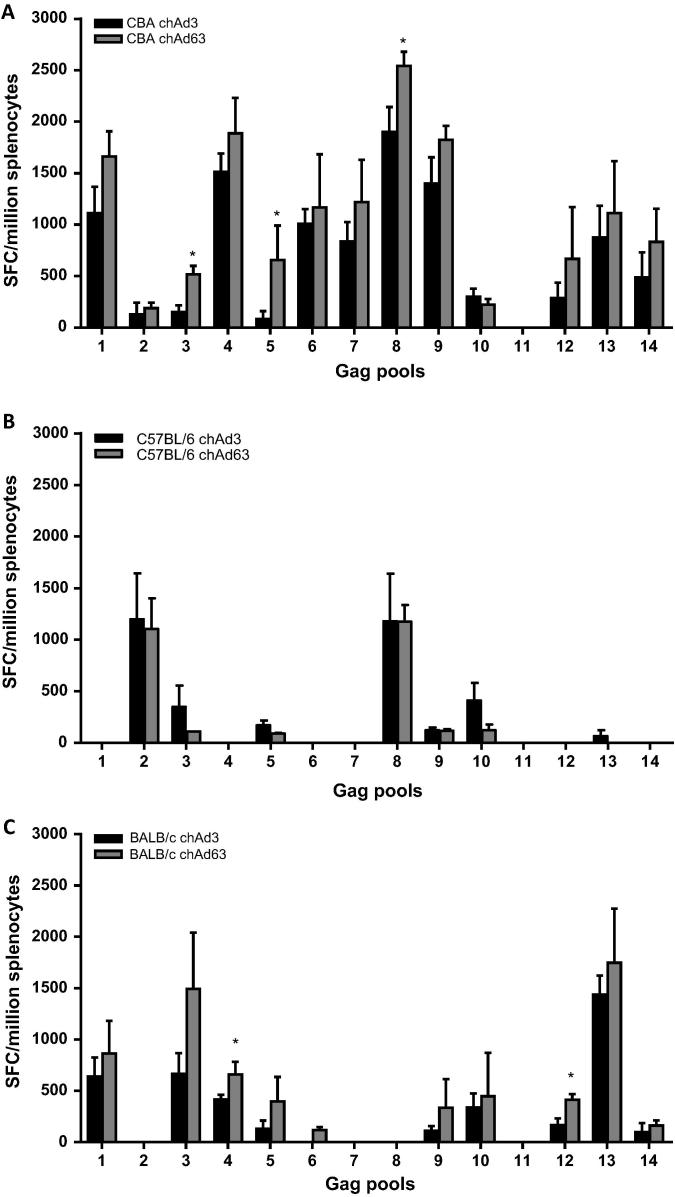
IFNγ responses to Gag peptide pools in different strains of mice. CBA (A), C57BL/6 (B) and BALB/c (C) mice were vaccinated with either 1 × 10^8^ IU of either ChAd3-GPN (black bar) or 1 × 10^9^ IU ChAd63-GPN (grey bar). Splenocytes were harvested and stimulated with the indicated gag peptide pools. IFNγ production was determined by ELISpot and the graphs represent the mean of 6 mice ± SD from 2 separate experiments. ^*^p < 0.05.

It is well documented that protective responses against HIV are Gag dominated [5], [7] and so we determined the immune response to the *gag* transgene in CBA, C57BL/6 and BALB/c strains of mice (Fig. 1). The magnitude and breadth of IFNγ responses seen in CBA (Fig.1A) and BALB/c (Fig.1C) mice were greater than those of C57BL/6 mice (Fig.1B). Unlike C57BL/6 mice where we saw responses to 7 out of 14 Gag peptide pools, CBA mice responded to 13 out of 14 peptide pools and BALB/c mice responded to 10 out of 14 peptide pools. In addition, some responses were significantly higher in ChAd63 vaccinated animals compared to ChAd3 vaccinated animals in CBA (peptide pools 3, 5 and 8) and BALB/c strains of mice (peptide pools 4 and 12) (Fig.1A and C).

### 3.2 No correlation between predicted and measured responses

**Fig. 2:**
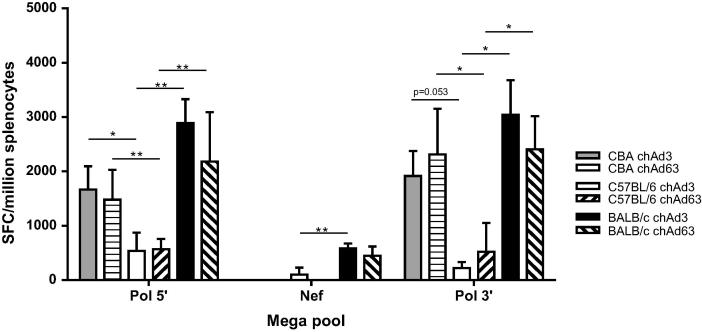
IFNγ responses to mega pools in different strains of mice. CBA (grey bar and horizontal hatched bar), C57BL/6 (open bar and left diagonal hatched bar) and BALB/c (closed bar and right diagonal hatched bar) mice were vaccinated with either 1 × 10^8^ IU of either ChAd3-GPN (solid bar) or 1 × 10^9^ IU ChAd63-GPN (hatched bar). Splenocytes were harvested and stimulated with the indicated mega pools. IFNγ production was determined by ELISpot and the graph represents the mean of 3 mice ± SD. ^*^p < 0.05 and ^**^p < 0.01.

To assess whether the magnitude and breadth of the response were due to more efficient epitope binding by some strains of mice, we investigated the binding prediction (http://www.mpiib-berlin.mpg.de/mpiib-cgi/MAPPP/binding) of the transgenes for H-2K restriction and chose scores above 16 as a cut off for efficient binding (Table 1). The results predict that BALB/c mice have the highest number of epitopes binding for all the transgenes followed by CBA mice and C57BL/6 mice. However, BALB/c mice did not have the highest frequency of responses to Gag as predicted (Fig.1C). We also determined whether the results in Table 1 matched the *in vivo* data for the *pol 5*′, *nef* and *pol 3*′ parts of the GPN transgene by assessing the immune responses in both ChAd3 and ChAd63 vaccinated CBA and BALB/c strains of mice. Responses to the megapools (all peptides for the protein present in a single pool) of the aforementioned transgenes were studied and our results showed that there were significantly higher responses to the Pol 5′ and Pol 3′ megapools in CBA and BALB/c strains of mice compared to C57BL/6 mice with both vaccinated vectors (Fig. 2). There were no responses to Nef in CBA mice with either vector despite the binding prediction, but there were responses to Nef in BALB/c mice with both vectors.

### 3.3 Transgene and vector responses differ between animal strains

**Fig. 3:**
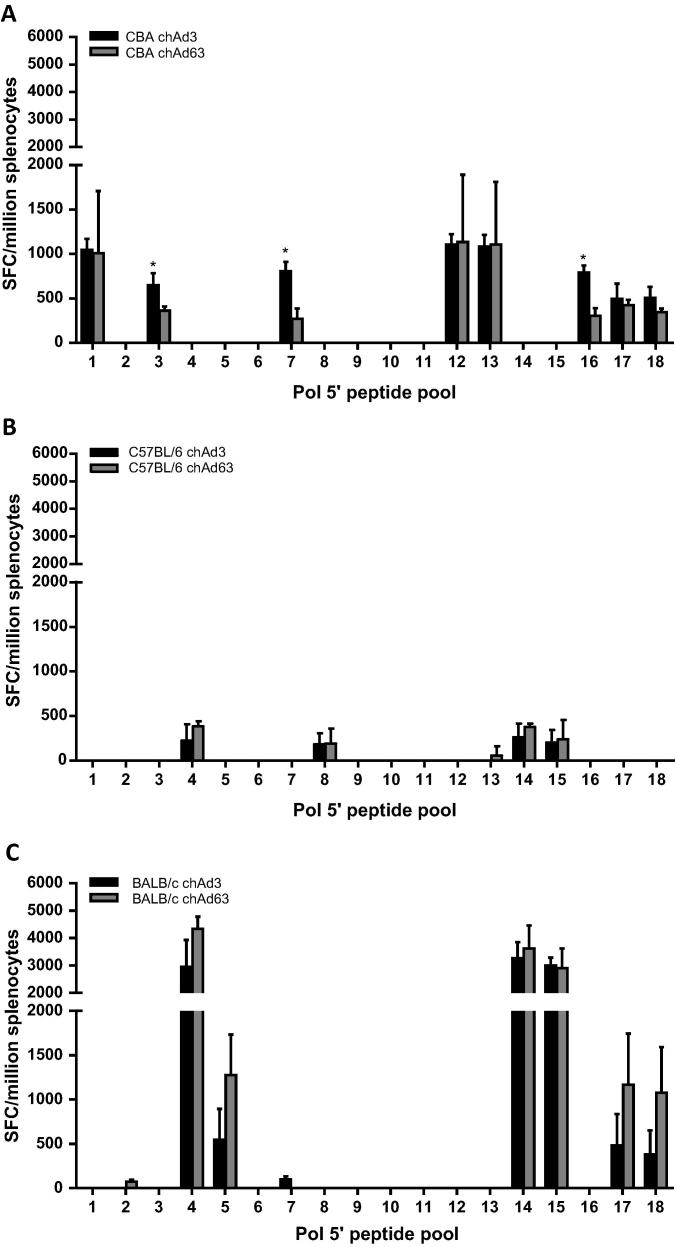
IFNγ responses to Pol 5′ peptide pools in different strains of mice. CBA (A), C57BL/6 (B) and BALB/c (C) mice were vaccinated with either 1 × 10^8^ IU of either ChAd3-GPN (black bar) or 1 × 10^9^ IU ChAd63-GPN (grey bar). Splenocytes were harvested and stimulated with the indicated Pol 5′ peptide pools. IFNγ production was determined by ELISpot and the graphs represent the mean of 3 mice ± SD. ^*^p < 0.05.

To investigate further the breadth and magnitude of the responses in the different strains, we vaccinated the strains of mice with either vector and analysed the IFNγ response to smaller peptide pools for each transgene element. With respect to Pol 5′, the C57BL/6 strain responded to 4 peptide pools with both vectors (pools 4, 8, 14 and 15) and one peptide pool with ChAd63 (pool 13) out of 18 peptide pools (Fig.3B), CBA mice responded to 8 peptide pools with both vectors (pools 1, 3, 7, 12, 13, 16, 17, and 18; Fig.3A) and BALB/c mice responded to 6 peptide pools with both vectors (pools 4, 5, 14, 15, 17, and 18) and one peptide pool with ChAd63 (pool 2) and one with ChAd3 (pool 7; Fig.3C).

**Fig. 4:**
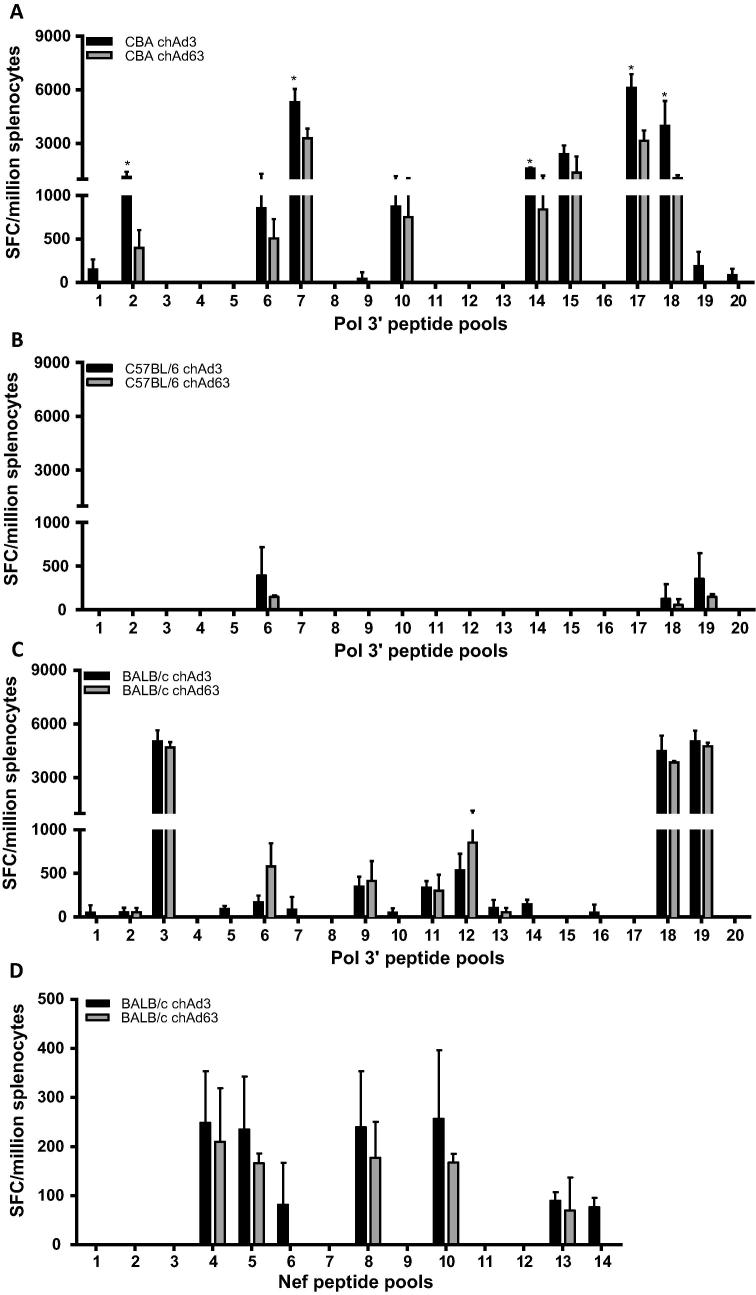
IFNγ responses to Pol 3′ and Nef peptide pools. CBA (A), C57BL/6 (B) and BALB/c (C and D) mice were vaccinated with either 1 × 10^8^ IU of either ChAd3-GPN (black bar) or 1 × 10^9^ IU ChAd63-GPN (grey bar). Splenocytes were harvested and stimulated with the indicated Pol 3′ (A–C) or Nef (D) peptide pools. IFNγ production was determined by ELISpot and the graphs represent the mean of 3 mice ± SD for graphs (A), (B) and (C) and graph (D) represent the mean of 6 mice ± SD from 2 separate experiments. ^*^p < 0.05.

Furthermore, in the CBA strain of mice, there were some significant differences in responses to peptide pools between ChAd3 and ChAd63 vaccinated animals (Fig.3A; peptide pools 3, 7 and 16). When we analysed the Pol 3′, we found that compared to the C57BL/6 strain, which responded to 3 out of 20 peptide pools with either vector vaccination (Fig.4B), CBA mice responded to 8 peptide pools with either vector vaccination (pools 2, 6, 7, 10, 14, 15, 17, and 18) and 4 peptide pools with only ChAd3 vaccination (pools 1, 9, 19, and 20; Fig.4A), and BALB/c mice responded to 9 peptide pools with either vector vaccination (pools 2, 3, 6, 9, 11, 12, 13, 18 and 19) and 6 peptide pools with only ChAd3 vaccination (pools 1, 5, 7, 10, 14 and 16; Fig.4C). Again, we saw that in the CBA strain of mice, there were significant differences in response to some peptide pools between ChAd3 and ChAd63 vaccinated animals (Fig.4A; peptide pools 2, 7, 14, 17 and 18).

With respect to Nef, BALB/c mice responded to 5 out of 14 peptide pools with either vector vaccination (pools 4, 5, 8, 10 and 13) and 2 peptide pools with only ChAd3 vaccination (pools 6 and 14; Fig.4D). Our previous work showed that C57BL/6 mice responded to 3 out of 14 peptide pools to Nef with only the ChAd3 vaccination. No responses were seen in CBA mice to the Nef peptide pools (data not shown).

### 3.4 Heat map of IFNγ responses to all peptide pools in different strains of mice

**Fig. 5:**
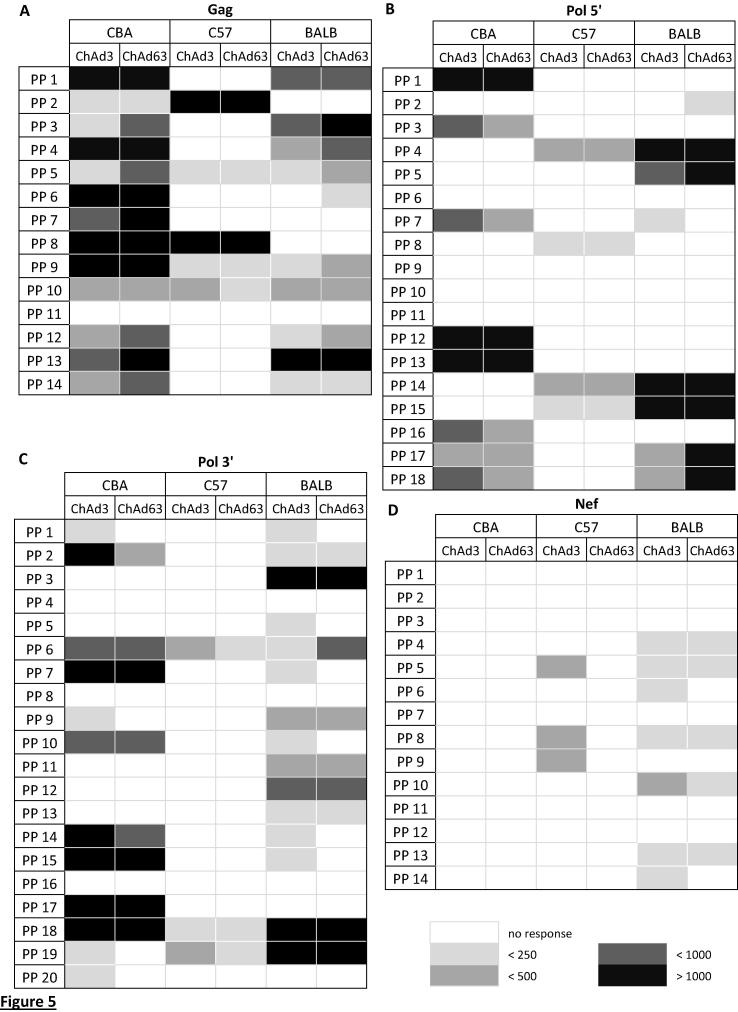
Heat map of IFNγ responses to all peptide pools in different strains of mice. Results from Fig. 1, Fig. 3, Fig. 4, Fig. 5 were collated into a heat map to show IFNγ responses for each strain of mouse either vaccinated with ChAd3 or ChAd63 against (A) Gag, (B) Pol 5′, (C) Pol 3′ and (D) Nef peptide pools (PP). The colour scheme is shown and corresponds to white as no response, light grey as less than 250 IFNγ producing cells (SFC), grey as less than 500 SFC, dark grey as less than 1000 SFC and black as more than 1000 SFC.

We collated the data into a heat map to compare all the responses between the strains of mice and vector vaccinations (Fig. 5). With Gag, responses to 3 peptide pools were common to all three strains of mice with both vector vaccinations (Fig.5A; peptide pools 5, 9 and 10), and 2 common peptide pools were observed to Pol 3′ (Fig.5C; peptide pools 6 and 18). There were no common peptide pools to Pol 5’ and Nef (Fig.5B and D). The map also showed that there were differing magnitudes of response to some common peptide pools between the strains of mice and that the C57BL/6 strain of mouse had the least diverse response to Gag, Pol 5′, Pol 3′ and Nef peptides.

Taken together these results showed that there are differing responses to HIV proteins in different strains of mice. Additionally, there are some significant differences between the different viral vectors for vaccination in CBA and BALB/c strains of mice.

## 4 Discussion

Assessment of an efficient prophylactic HIV vaccine involves identifying broad immune responses to highly conserved HIV proteins. The literature indicates that the majority of animal experiments to evaluate immunogenicity have predominantly taken place in mice with the preferred strain being C57BL/6. However in our hands we found that in response to a ChAd vaccine with HIV proteins, the C57BL/6 mice had a limited range of T cell responses [9]. We therefore decided to determine whether there were differences in the immune response to the HIV transgenes with ChAd vaccination in different strains of mice.

We decided to use CBA, C57BL/6 and BALB/c mice as they have different H-2K and H-2D restrictions with CBA mice being K^k^ and D^k^ restricted, C57BL/6 being K^b^ and D^b^ restricted, and BALB/c being K^d^ and D^d^ restricted. Although this did not cover the whole range of H-2 restrictions within the strains of mice, it did provide three distinct genetic backgrounds to test whether there were any differences in immune responses to the same HIV transgenes. Furthermore, it provided the opportunity to determine whether the vectors used in the vaccination protocol impacted on the immune response.

Unsurprisingly, we found that the responses to the transgene constituents were different between the strains of mice but interestingly the C57BL/6 strain showed the most limited immune response with ChAd3 or ChAd63 vectors. The broadest and highest responses to the Gag, Pol 5′ and Pol 3′ were seen in the CBA and BALB/c strain of mice (Fig. 1, Fig. 2, Fig. 3, Fig. 4) and of these two strains, only BALB/c mice responded to the *nef* transgene in both ChAd3 and ChAd63 vaccinations (Fig.4D).

The ability of CBA and BALB/c mice to respond so dynamically to the transgene may in part be due to their haplotype restrictions. Analysis of the predicted epitope binding data showed that both CBA and BALB/c mice were able to bind 8-mer, 9-mer and 10-mer peptides to H-2K while C57BL/6 mice were only predicted to be able to bind 8-mer peptides. Indeed, structural analysis of the different H-2K restrictions show that they bind peptides in different regions resulting in different presentation to T cells [11], [12], [13]. This restriction in binding may result in lowered immune responses to longer peptides in C57BL/6 mice which may be a result of differential proteasomal processing. However, we did not test this hypothesis using peptides of different lengths.

Furthermore, the ability of BALB/c mice to respond at a higher magnitude to some peptide pools and also to the *nef* transgene may, in part, be due to the fact that in addition to H-2K and H-2D, BALB/c mice also have a third allele H-2L which CBA and C57BL/6 mice do not (https://www.bdbiosciences.com/documents/mouse_alloantigens_chart.pdf). However, ChAd3 vaccinated C57BL/6 mice, were able to respond to some peptide pools to the *nef* transgene though the responses were very low, which suggests that while the H-2L may confer some epitope binding of Nef peptides it is not the only allele to do so. Speir et al. [14] have shown that H-2L^d^ peptides show a similar binding anchorage to H-2K^b^ (C57BL/6 mice) which suggests that the anchorage similarity may bind similar peptides within Nef.

The lack of responses to the *nef* transgene in CBA mice may to some extent be due to the dominant responses seen to the *gag* and *pol* transgenes, which may mask the much lower responses that may be present. However, the lack of Nef responses may be detrimental following a viral challenge as Nef responses are shown to be linked with lower viral loads [15]. It would have been interesting to see if the CBA mice were able to mount responses to the *nef* transgene in a single transgene vector, but this was, however, beyond the scope of this study.

In addition to the H-2 restriction for class I, mice also have class II alleles, I-A and I-E. While both CBA and BALB/c mice have both I-A and I-E alleles, C57BL/6 mice only have I-A alleles. This suggests that in C57BL/6 mice there may be limited class II presentation which may influence CD4 T cell activation and CD4 T cell help resulting in the lowered responses to HIV transgenes observed in this strain of mice. Indeed, the importance of CD4 T cells in immune responses and CD8 T cell activation is well documented [16], [17] and suggests that C57BL/6 mice may not be an ideal model if CD4 T cell activation is required. Taken together, the absence of the H-2L allele and the I-E allele in C57BL/6 mice may influence antigen presentation of our HIV transgenes and may explain to some extent the lowered IFNγ responses detected compared to the other two strains used in this study.

In addition to investigating the immune responses to HIV transgenes we also determined whether the vaccine vector influenced the response in the different strains. We have previously shown that in the C57BL/6 strain of mice there was no difference in IFNγ production between the two vectors, ChAd3 and ChAd63, when dosing was normalised. However, we observed some significant differences in responses to some peptide pools to the *gag* transgene in CBA and BALB/c mice vaccinated with ChAd63. To have comparable results between the two vectors in our C57BL/6 mouse model, we had to inject a 10-fold higher concentration of ChAd63, so these significant differences seen in the CBA and BALB/c mouse model may be simply due to an increase in the vaccine vector. Indeed, on analysis of the data for the *gag* transgene, most responses to peptide pools were higher in the ChAd63 vaccinated CBA and BALB/c animals though not all were significant. However, analysis of the other transgenes showed that in CBA mice, there were significant differences in some responses to the *pol* transgenes in ChAd3 vaccinated animals and no differences between the vectors for BALB/c mice. Consequently the higher responses seen in ChAd63 vaccinated animals cannot simply be explained by an increase in dose. As both vectors use the CAR receptor for entry into cells [4] it is unlikely that receptor expression can solely explain any differences observed. These results suggested that immune responses to HIV proteins are dependent on a combination of the vector used, the transgene of interest and the haplotype of the mouse model being employed. We have not statistically quantified the level and duration of expression of GPN from each vector in the different mouse strains and expression of antigen transcripts was seen by other investigators to vary between ChAd3 and 63 vectors expressing SIV-gag [18].

It has been shown previously that both ChAd3 and ChAd63 bearing a dominant HIV gag peptide induced a low frequency of IFNγ only producing T cells but a high percentage of polyfunctional T cells [1]. Our experiments did not include investigation of the polyfunctionality of the responding cells. Furthermore, variation of type I IFN responses between different rAd vectors was reported and correlated to immunogenicity. Different mouse strains have been shown to produce different responses to pathogenic challenge and strain specific immunogenetic differences have been described and were not limited to differences in MHC [19]. Indeed, the innate immune response to the vaccine may have influenced the IFNγ response observed in our experiments. Sellers et al. [19] discuss the differences in innate immune receptor complexes between strains of mice and how they may influence the adaptive response. An example is the expression of Killer cell lectin-like receptors on NKT cells which are able to recognise MHC class I peptide expression resulting in the production of IFNγ and their expression is highly divergent between strains of mice. Furthermore the authors identify that CBA and C57BL/6 mice have a Th1 bias to pathogens while BALB/c mice are TH2 biased. Taken together, the differences observed in our study may be due to a number of different factors such as innate immunity and MHC alleles and identifies the complexity between proteins and immune responses.

Collating the data into a heat map helped to identify common peptide pools between the three strains of mice and despite the difference in haplotypes there were a limited number of common peptide pools for some of the transgenes (Fig. 5). We did not determine the individual peptide within the common pools that the strains of mice responded to so it is not possible to know if the different strains responded to the same peptide or a variety of peptides present within the same pool.

Taken together our results showed that there were differences in immune responses to the same transgenes between different strains of mice and importantly that there were, in some cases, significant differences in responses to different vectors in some strains of mice. As C57BL/6 mice tend to be the mouse strain of choice, our results suggested that we, along with other groups, may have underestimated the potential of our vaccine candidate due to lower immune responses. Dicks et al. [20] have shown that that immune responses induced by ChAds differ between laboratory animals and a target species but this study has highlighted the importance in the choice of mouse strain in determining immune responses and that less than favourable results may not be indicative of the product being tested but may be due to the genetics of the mouse being employed.

## Conflict of interest

SC is the co-inventor of the following patents: “Chimpanzee Adenovirus Vaccine Carriers” WO 2005/071093 A2, “Hepatitis C Virus Nucleic Acid Vaccine” WO 2006/133911 A2 and “Simian Adenovirus Nucleic Acid-and Amino Acid-Sequences, Vectors containing same and uses thereof” WO 2010/086189 A3. All mentioned patents are under control of GlaxoSmithKline SA and no significant financial support for this work has influenced its outcome. All other authors have no conflict of interest related to this work.

## Figures and Tables

**Table 1:** Number of possible epitopes binding H-2K alleles in CBA, C57BL/6 and BALB/c strains of mice. Sequence data for the *gag*, *pol 5*′, *nef* and *pol 3*′ transgenes were submitted to the prediction binding program MAPPP (http://www.mpiib-berlin.mpg.de/mpiib-cgi/MAPPP/binding) specifying mouse haplotype and peptide length. Binding scores above 16 were tabulated against the strain of mouse for each transgene and each vaccine vector.

	Strain of mouse		
CBA	C57BL/6	BALB/c
Gag	14	7	26
Pol 5′	12	18	22
Nef	8	3	18
Pol 3′	29	6	48

## References

[1] Quinn K.M., Da Costa A., Yamamoto A., Berry D., Lindsay R.W., Darrah P.A. (2013). Comparative analysis of the magnitude, quality, phenotype, and protective capacity of simian immunodeficiency virus gag-specific CD8+ T cells following human-, simian-, and chimpanzee-derived recombinant adenoviral vector immunization. J Immunol.

[2] Dicks M.D., Spencer A.J., Edwards N.J., Wadell G., Bojang K., Gilbert S.C. (2012). A novel chimpanzee adenovirus vector with low human seroprevalence: improved systems for vector derivation and comparative immunogenicity. PLoS ONE.

[3] Barouch D.H. (2010). Novel adenovirus vector-based vaccines for HIV-1. Curr Opin HIV AIDS.

[4] Colloca S., Barnes E., Folgori A., Ammendola V., Capone S., Cirillo A. (2012). Vaccine vectors derived from a large collection of simian adenoviruses induce potent cellular immunity across multiple species. Sci Transl Med.

[5] Kiepiela P., Ngumbela K., Thobakgale C., Ramduth D., Honeyborne I., Moodley E. (2007). CD8+ T-cell responses to different HIV proteins have discordant associations with viral load. Nat Med.

[6] Barouch D.H., Stephenson K.E., Borducchi E.N., Smith K., Stanley K., McNally A.G. (2013). Protective efficacy of a global HIV-1 mosaic vaccine against heterologous SHIV challenges in rhesus monkeys. Cell.

[7] Sacha J.B., Chung C., Rakasz E.G., Spencer S.P., Jonas A.K., Bean A.T. (2007). Gag-specific CD8+ T lymphocytes recognize infected cells before AIDS-virus integration and viral protein expression. J Immunol.

[8] Borthwick N., Ahmed T., Ondondo B., Hayes P., Rose A., Ebrahimsa U. (2014). Vaccine-elicited human T cells recognizing conserved protein regions inhibit HIV-1. Mol Ther.

[9] Herath S., Le Heron A., Colloca S., Bergin P., Patterson S., Weber J. (2015). Analysis of T cell responses to chimpanzee adenovirus vectors encoding HIV gag-pol-nef antigen. Vaccine.

[10] Search P. <http://www.ncbi.nlm.nih.gov/pubmed/?term=mouse+and+HIV>.

[11] Kellenberger C., Roussel A., Malissen B. (2005). The H-2Kk MHC peptide-binding groove anchors the backbone of an octameric antigenic peptide in an unprecedented mode. J Immunol.

[12] Mitaksov V., Fremont D.H. (2006). Structural definition of the H-2Kd peptide-binding motif. J Biol Chem.

[13] Zhang W., Young A.C., Imarai M., Nathenson S.G., Sacchettini J.C. (1992). Crystal structure of the major histocompatibility complex class I H-2Kb molecule containing a single viral peptide: implications for peptide binding and T-cell receptor recognition. Proc Natl Acad Sci USA.

[14] Speir J.A., Garcia K.C., Brunmark A., Degano M., Peterson P.A., Teyton L. (1998). Structural basis of 2C TCR allorecognition of H-2Ld peptide complexes. Immunity.

[15] Adland E., Carlson J.M., Paioni P., Kloverpris H., Shapiro R., Ogwu A. (2013). Nef-specific CD8+ T cell responses contribute to HIV-1 immune control. PLoS ONE.

[16] Younas M., Psomas C., Reynes J., Corbeau P. (2016). Immune activation in the course of HIV-1 infection: causes, phenotypes and persistence under therapy. HIV Med.

[17] Zhang S., Zhang H., Zhao J. (2009). The role of CD4 T cell help for CD8 CTL activation. Biochem Biophys Res Commun.

[18] Quinn K.M., Zak D.E., Costa A., Yamamoto A., Kastenmuller K., Hill B.J. (2015). Antigen expression determines adenoviral vaccine potency independent of IFN and STING signaling. J Clin Invest.

[19] Sellers R.S., Clifford C.B., Treuting P.M., Brayton C. (2012). Immunological variation between inbred laboratory mouse strains: points to consider in phenotyping genetically immunomodified mice. Vet Pathol.

[20] Dicks M.D., Guzman E., Spencer A.J., Gilbert S.C., Charleston B., Hill A.V. (2015). The relative magnitude of transgene-specific adaptive immune responses induced by human and chimpanzee adenovirus vectors differs between laboratory animals and a target species. Vaccine.

